# Supplementary material for: Interplay of valley polarized dark trion and dark exciton-polaron in monolayer WSe2
Source: Nat Commun. 2023 Sep 13;14:5657. doi: 10.1038/s41467-023-41475-4 (PMC10500002; doi:10.1038/s41467-023-41475-4)
Supplement: Supplementary file 1 — Supplementary Information [file 41467_2023_41475_MOESM1_ESM.pdf]

Supplementary Information for

"Interplay of valley polarized dark trion and dark exciton-polaron in monolayer WSe_2"

Xin Cong[1], Parisa Ali Mohammadi[1], Mingyang Zheng[1], Kenji Watanabe[2], Takashi Taniguchi[3], Daniel Rhodes[4], Xiao-Xiao Zhang[1]*

[1]*Department of Physics, University of Florida, Gainesville, Florida, USA*

[2]*Research Center for Functional Materials, National Institute for Materials Science, 1-1 Namiki, Tsukuba, Japan*

[3]*International Center for Materials Nanoarchitectonics, National Institute for Materials Science, 1-1 Namiki, Tsukuba, Japan*

[4]*Department of Materials Science and Engineering, University of Wisconsin Madison, Madison, Wisconsin, USA*

*Corresponding author: xxzhang@ufl.edu

1. Spectroscopy setup

Figure S1a shows the schematics of the Fourier plane imaging setup for the energy- and momentum-resolved PL measurements. An example of a typical spectrum obtained in this method can be seen in Fig. S1b, which demonstrates the contrast between the bright and dark exciton emission. The white dashed line outlined the in-plane emission contribution of the dark exciton. The obtain the dark exciton PL, we take the spectrum at the edge of the CCD (large momentum) and subtract the bright emission component taken at the center of the CCD (zero momentum) multiplied by a constant factor.

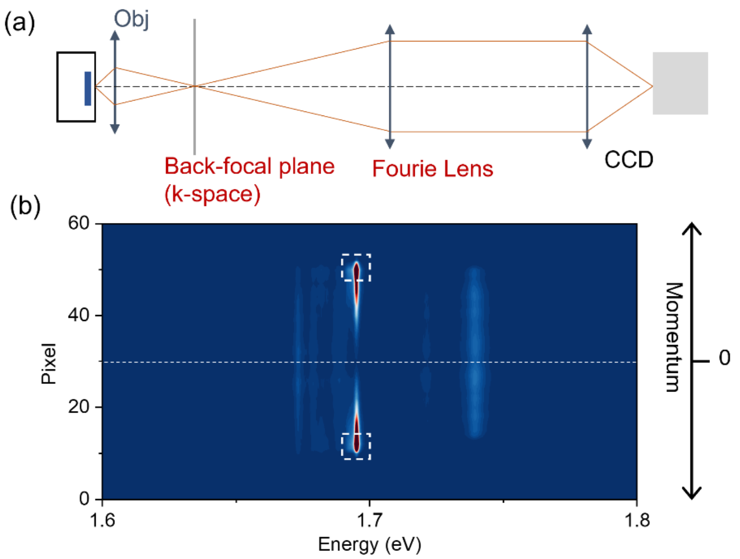

Figure S1. (a) Schematics for the Fourier plane imaging optical setup. **(b)** A typical Fourier plane imaging results of monolayer WSe_2 PL. The white dashed line indicates emission with zero in-plane momentum. Bright excitons have an in-plane dipole and a stronger emission along the zero momentum, while dark excitons (indicated by white dashed line squares) have strong emission with larger momentum.

2. Detailed fitted results of dark trions

The results shown in the main text Fig. 2 are obtained by fitting the dark trions with two Voigt profiles at different fluences, magnetic fields, and temperatures. Figure S2 shows the fitted integrated PL intensity and linewidths of D1+ and D2+ of the spectrum shown in Fig. 1d. The increase and decrease of $D1^+$ and $D2^+$ amplitudes can be clearly seen when going across V_c. As shown in Fig. 2S(b), $D1^+$ goes through a slight decrease in linewidth while $D2^+$ shows a significant increase. The fitted linewidth for $D2^+$ between -4V to -3V is inaccurate because $D2^+$ evolved into a broad spectral shoulder with large linewidth and relatively small amplitudes. Additionally, Fig. S3 shows the crossover voltage extracted from the linewidth broadening (across 10% increase) in Fig. 2d. The exact value is slightly different from Fig. 2e, which depends on the choice of the "threshold" broadening percentage. However, the fitted linear slope of 9 $\frac{meV}{\mu J \cdot cm^2}$ agrees well with the one obtained in Fig. 2e, which confirms that the origin of spectral changes in $D1^+$ and $D2^+$ is related to the same critical doping density, which depends on the exciton density.

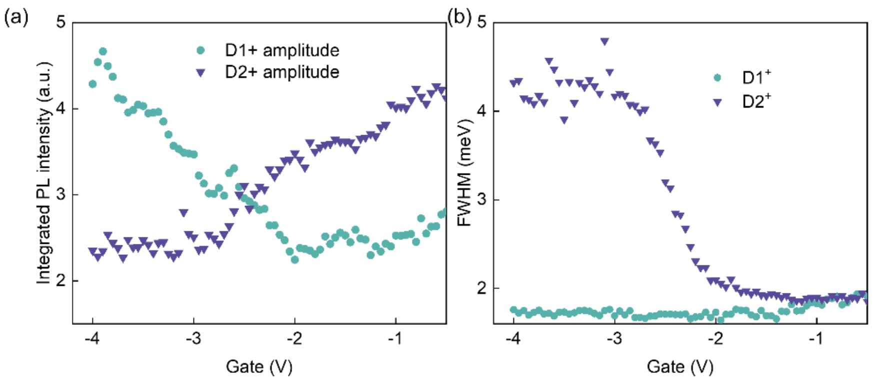

Figure S2 (a) Fitted amplitudes of $D1^+$ and $D2^+$ with $2.5 \mu J/cm^2$ fluence 1.88 eV excitation, at 4K and under a 9T out-of-plane magnetic field. The peaks are fitted with a Voigt profile. **(b)** The corresponding FWHM of the two dark trions from the same measurement.

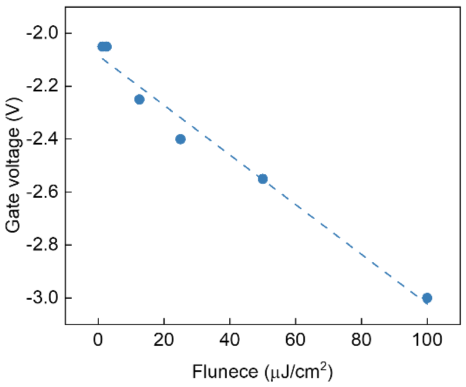

Figure S3. The crossover voltage as extracted from the linewidth broadening of $D2^+$, as described in the main text and the SI Section 2.

3. Valley polarization of dark trions and intervalley coupling

The valley compositions of different excitonic species are examined with both PL and reflection contrast measurements with circularly polarized excitation. Dark exciton PL is z-polarized in this detection method and therefore cannot be used to distinguish the valley polarization. With circularly polarized excitation, valley-polarized dark excitons can be created and maintained before emission, which gives rise to different PL amplitudes. Fig. S4a shows the reflection contrast of neutral bright excitons with different circularly polarized whitelight reflection contrast. σ- excitation corresponds to the higher energy excitonic state and K' valley bright exciton (see the band schematics in Fig. S4c and Fig. 1 in the main text). In the p-doped region as shown in Fig. S4b, the Zeeman split dark trions $D1^+$ is favored with K valley excitation, and $D2^+$ is favored with K' valley excitation. This can be understood by considering the optically-excited electron relaxation pathway as shown in Fig. S4c: the electron is excited into the K' valley upper conduction band and goes through a rapid intervalley scattering into the K valley lower conduction band, which contributes to the formation of the $D2^+$ K valley dark trion state. Overall, the valley-polarized excitation creates an imbalance in electron carriers, while the hole side imbalances are less significant due to the presence of free carrier doping.

With circularly polarized excitation, we can investigate the intervalley coupling between the different dark exciton polaron. Fig. S5 shows the percentage linewidth broadening of $D2^+$ as a function of gate (all taken at dilute exciton limit) when polarized excitation. With linearly polarized excitation, both $D1^+$ and $D2^+$ are created, while σ- excitation creates mostly $D2^+$ excitons. There is a ~0.2V delay onset of the $D2^+$ linewidth broadening with σ- excitation , which indicates that the $D2^+$ spectral changes in region (ii) is not only determined by the free carriers but also by the presence of $D1^+$.

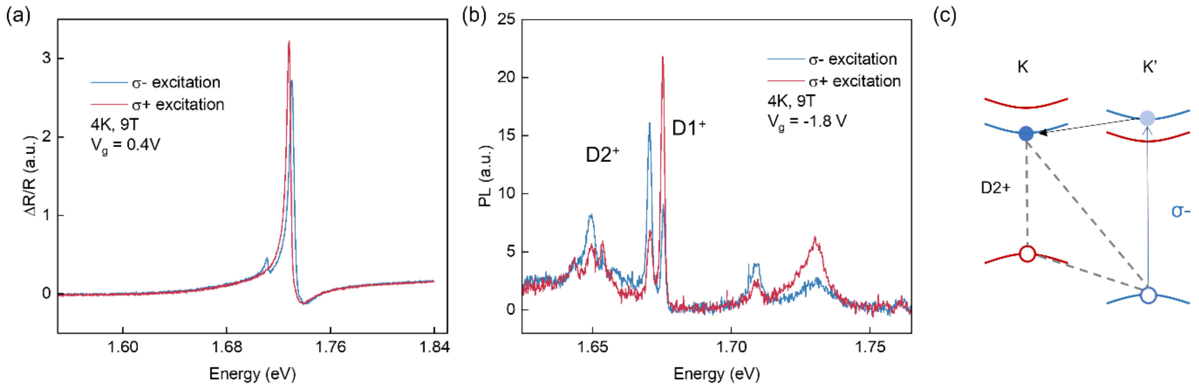

Figure S4. **(a)** Reflection contrast measurements with polarized excitation close to the charge neutral point. σ+ excitation creates K valley excitons bright, which has lower energy due to the conduction and valence bands Zeeman splitting. **(b)** Dark exciton PL at the p-doped region. σ+ excitation favors the formation of $D1^+$, which corresponds to the K' valley dark exciton that interacts with holes in the K valley valence band. σ- excitation favors the formation of $D2^+$, which is the K valley dark trion state. **(c)** With σ- excitation, K' valley bright excitons are created. The optically-excited conduction band electrons go through intervalley scattering to the K valley bottom conduction band, which forms the $D2^+$ trion state.

4. Doping dependence of bright states

The bright exciton doping dependence at 4K with a 9T out-of-plane magnetic field can be seen in Fig. S6 a&b. The Zeeman splitting for the bright neutral exciton (near 1.73 eV) and bright trion (near 1.71 eV) can be resolved with circular polarization and consistent with previous reports but cannot be directly

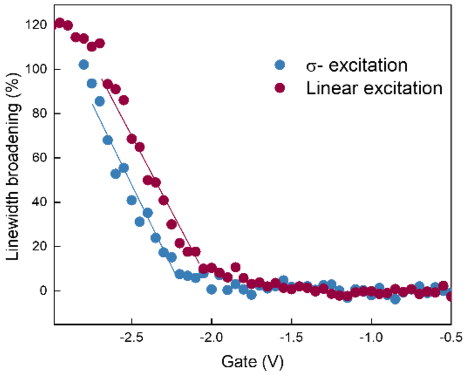

Figure S5. The D2$^+$ percentage linewidth broadening with σ- and linear excitation conditions respectively. The broadening onset with σ- excitation is delayed by 0.2V.

observed here due to their spectral linewidth. For the zoomed-in 1V to -4V range, the neutral bright exciton and bright trion PL peaks showed energy blueshift and redshift in the p-doping range that are consistent with the reflection contrast spectra (Fig. 3 in the main text). Notably, the redshift of the bright trion, which is dominated by the K' valley intervalley trion in amplitude, is also consistent with the gating dependence of D1$^+$.

The phonon replicas of the dark trions can also be observed at lower energies. The Γ_5 phonon replicas of D1$^+$ and D2$^+$ are at 1.655 eV and 1.650 eV, respectively at low doping. The D2$^+$ phonon replica shows a higher intensity than that of the D1$^+$, which may come from the imbalance of hole carriers and thermal relaxations. These phonon replicas energy shifts also show the consistent two-segment doping dependence as the D1$^+$ and D2$^+$ discussed in the main text. Notably, the D2$^+$ phonon replica does not show a significant linewidth broadening in the region (ii), and the energy redshift as a function of gate eventually increases to be comparable with D1$^+$ and D1$^+$ phonon replicas at even higher doping levels. This is also consistent with the expected behavior when the carrier densities in both valleys are above the critical density.

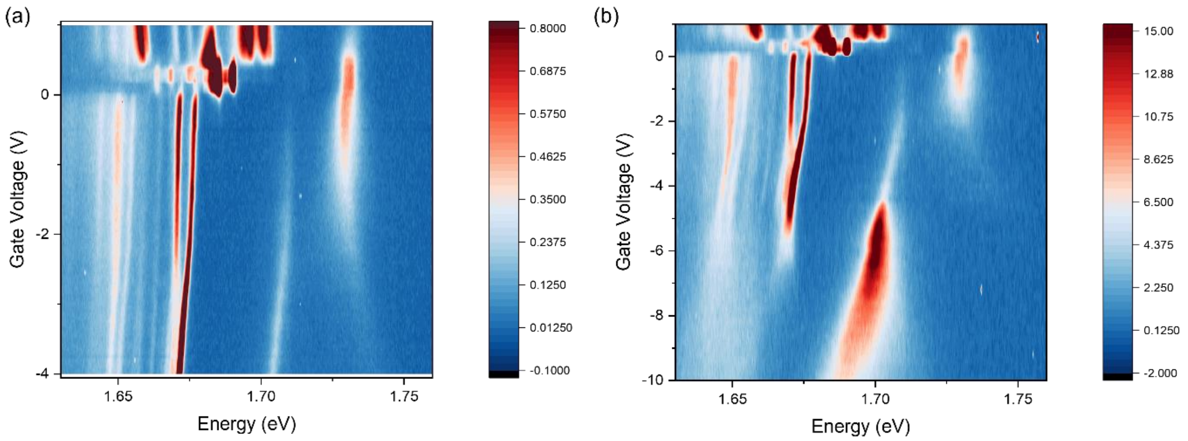

Figure S6. Gate-dependent PL, including bright, dark and dark phonon replica signals, for a zoomed-in fine scan range **(a)** and of higher doping levels **(b)**.

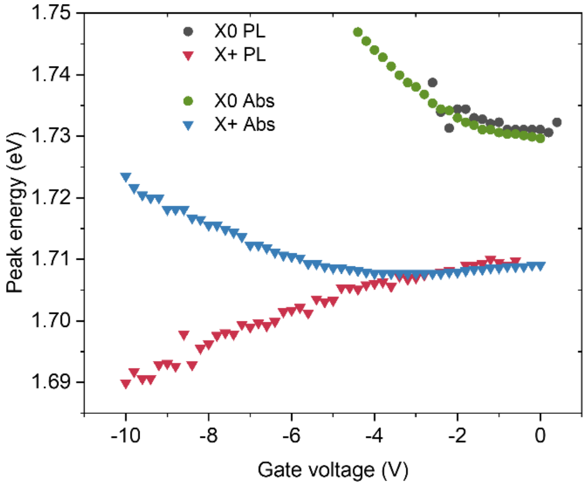

Figure. S7. Comparison between peaks energies of the bright neutral exciton (X0) and bright p-type trion (X+) as measured from PL spectroscopy and reflection contrast spectroscopy (absorption). The plotted measurements were done at 4K, 0T.

In Fig. S7, we compare the PL and absorption signals of the bright excitonic states (neutral exciton and p-type trion) measured at 4K, 0T. The PL and absorption of the bright trion state showed an energy splitting from ~ -3V, which corresponds to a doping density of $2.1 \times 10^{12}/cm^2$, and therefore a density of ~ $1 \times 10^{12}/cm^2$ in each of the valleys. The results of the bright excitons and trions are consistent with previous reports, and the energy splitting between the PL and absorption has been considered as a signature of the roton effects[1,2].

5. Interactions between bright and dark exciton polaron

Fig. S6b shows the PL gate dependence at higher doping. At sufficiently higher doping levels ($V_g < -5V$), the dark trions intensity decreases while the bright trion PL emission intensity increases. This can be qualitatively understood by considering the different dynamics of the bright and dark exciton polaron. Bright exciton polaron oscillator strength increases, and radiative decay becomes more efficient at high doping. The relaxation channels of optically-excited carriers into the bright state emission become a competing channel compared to the slow dark state emission and can introduce such amplitude transfer between the bright and dark polaron states. The $D1^+$ exciton lifetime as a function of gate voltage also supports such a picture. As shown in Fig. S8, the rise and decay of the D1+ remained roughly the same below -0.5V to -4V. Within -4V to -6V, there is a significant decrease of the decay lifetime from ~1 ns to 0.75ns, which occurs as the emission amplitude transfer from dark to bright exciton polaron states.

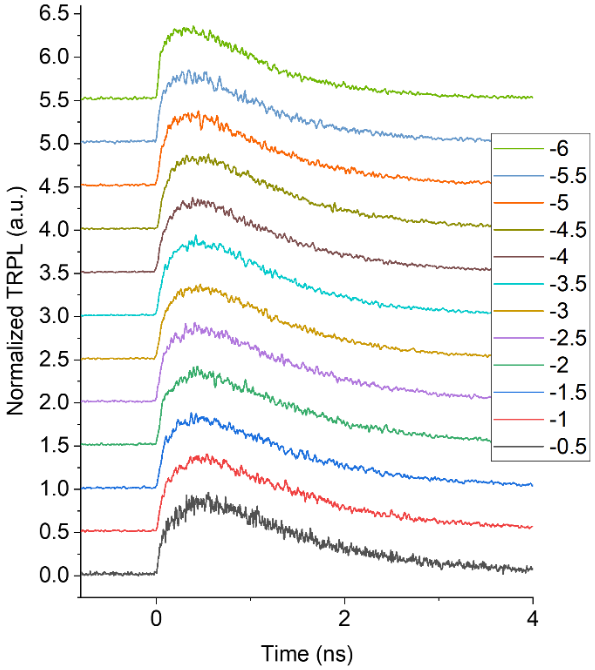

Figure S8. Time-resolved PL of the $D1^+$ state at different gate voltages, from -0.5 V to -6V. The rise times are around 200ps, and the decay times show an obvious decrease when $V_g < -4V$.

Suppelementray References:

1 Cotlet, O., Wild, D. S., Lukin, M. D. & Imamoglu, A. Rotons in optical excitation spectra of monolayer semiconductors. *Physical Review B* **101**, 205409, doi:10.1103/PhysRevB.101.205409 (2020).

2 Liu, E. *et al.* Gate-tunable exciton-polaron Rydberg series with strong roton effect. *arXiv preprint arXiv:2006.04895* (2020).
